# Two Cases of Sloughing Ulceration

**Published:** 1826-08

**Authors:** 

**Affiliations:** St. Thomas's Hospital


### SLOUGHING ULCERATION.

*Two Cases of Sloughing Ulceration.* Treated at St. Thomas's Hospital, by Mr. Travers.

### I. *Case of Sloughing Ulceration, in which the application of undiluted Nitric Acid was attended with success.*

September 30th, 1824.—Anne Skinner, aged seventeen, of delicate appearance, sallow complexion, light eyes and hair; stated that she had been on the town three months, and had resided in Swan-alley, St. Catherine's. While following this course of life, she was in the habit of indulging in the free use of spirits. She was admitted with a small irregular sloughy ulcer on each side of the perineum, which she said had existed for three weeks; during which time she had taken no medicine, although her health had suffered considerably.

℞. Mist. Camph. ʒxj.; Sp. Æther. Nitr. ʒj. M. ter die sumend.

Pulv. Opii, gr. j. nocte et mane.—Apply to the sores, Epithem. Opiat.

October 11th.—Under the above plan of treatment, the sores speedily assumed a healthy aspect. The ulcers are now clean, but the granulations indolent.

Dec. Cinchonæ, ʒx.; Tr. Ejusd. ʒj.; Ext. Ejusd. ℈ss. ter die.

Vini Rubri, ℥iv. in dies. Continue Epith. Opiat.

19th.—Sloughing process again commenced on the 15th, and has rapidly extended. The slough occupies the whole perineum, extending to the nates on the right side, from the tuber ischii to the level of the centre of the labia, which are much swollen and inflamed. On the left side the slough is similar to the other, but only half the size. The sloughs are of a black colour, and discharge a thin fetid ichor: their circumference is very tumid and inflamed, and the edges are white, everted, and irregular. She lies constantly moaning from severe burning pain, which deprives her of rest. The whole surface of the body and extremities is covered with a scaly copper-coloured eruption, which is accompanied with troublesome itching. Countenance anxious; pulse 120, firm and full; tongue furred, white, and moist; great thirst; bowels confined.

V.S. ad ℥xij.—Mist. Camph. ℥jss. c. Sp. Æther. Nitr. ʒj. ter die.
Pulv. Opii, gr. j. quater in die.—Vin. Rubri, ℥vj. quotidie. Ol. Ricini, p. r. n.
Fom. Papav. c. Cataplasm Cerevis., under which the Epithem. Opiat. is to be applied on lint.

20th.—Slept some hours, and continues drowsy. Complains more of pain. Pulse 120, quick, small, and hard; bowels confined; less inflammation at the edges of the slough, which has not spread.

Catap. Theriac. Lond.

21st.—Slept tolerably well; bowels opened last night, and again this morning; pulse 125, fuller and stronger; tongue less furred; appetite bad. The slough has spread a little on the right side; a small portion, at the verge of the anus, is inflamed and vesicated. Pain rather less acute.

22d.—Half the vesicated portion has sloughed. Increased inflammation of the labia, the external edges beginning to slough. Appetite improved. In other respects as before.

Mutton chop daily.—Repetantur medicamenta, &c.

23d.—Labia sloughing more rapidly. The slough has separated from the perineum and the left side of the nates, leaving the surface covered by a viscid tenacious pus; edges swollen and everted, not acutely inflamed. The undiluted Nitric Acid has been freely applied over the whole wound with a pencil-brush, working to the bottom of the slough, which was very dense and resisting. Two grains of opium were administered immediately. In twenty minutes she was free from pain.

24th.—Slept several hours. Swelling so much diminished that the wound appears one-third less: the edges are pale and inverted. Inflammation of labia subsided; still feels smarting pain. Little or no variation in pulse, tongue, or general symptoms.

Six P.M.—Nitric acid again applied, and opium repeated. Was "quite easy" in ten minutes.

27th.—The slough has separated from the left side.

November 2d.—The slough is now completely thrown off, leaving a florid granulating surface, which suppurates copiously. At the back part of the right thigh, the wound is an inch in depth, exposing the muscles for three inches, and becoming gradually more shallow: it extends two inches higher on the nates. On the left side, the wound occupies a circle two inches in diameter, connected with that on the opposite side by the perineum, the whole of which is involved. The external half of each labium is also destroyed. She has smarting pain in the wound, more severe when it is uncovered. The countenance has lost its anxious expression, and the complexion is clearer. Appetite better; pulse 100, moderately strong and full; tongue clean and moist; some thirst; bowels regular; sleeps well.

> Repetantur medicamenta. Discontinue treacle poultice, and apply black wash and linseed poultice.

19th.—Sore, which was healing rapidly, again become indolent.

> Applic. Ung. Terebinth.—Cerev. ℔j. in die.

22d.—The wound has been gradually healing, but her appetite is bad. She was removed to a clean ward on the 10th.

> ℞. Infusi Rosæ, Infusi Cascar. āā ℥v.; Acid. Sulph. dil. m. x.; Tr. Cinch. C. ℥j. M. ter die sumend.—Meat daily.

February 8th.—The improvement, though progressive, has been slow. From her confined position in the bed, her knees have become contracted.

> Hydr. c. Creta, gr. iv.; Hydr. Submur. gr. j. omni nocte.

Under the above plan she continued to improve, and by March 8th the sore was quite healed; but, on account of the contraction of the knees, she continued in the hospital till the 20th, at which time the free motion of the joints was not entirely restored. She has since enjoyed good health, and has got married.

II. *Case of Phagedenic Ulcer of the Genitals, in a Male.*

September 1st, 1825.—John Hamilton, ætat. eighteen, of fair complexion, light eyes and hair, and sickly appearance; by trade, apprentice to a joiner; accustomed to regular habits, and, according to his own account, having all his life enjoyed good health, up to the period of his present illness. Was admitted yesterday with "inflamed phymosis, and a sloughy sore surrounding the extremity of the prepuce." He immediately took a dose of Mist. Sennæ C., and had a poultice applied to the parts, which he was directed to keep supported against the abdomen.

3d.—States that, about three months since, (after connexion with a female in Swan-alley,) he had scalding and a slight discharge from the urethra; followed, in a few days, by two or three small sores on the inside of the prepuce, which had been in a state of phymosis ten days, but had begun to swell and become painful three days only prior to his admission. On examination this morning, the prepuce and integuments of the penis were found very acutely inflamed, with considerable swelling, vivid redness, acute pain, and intense heat; accompanied by great constitutional irritation, marked by a white furred tongue, with thirst, pain of head, heat of skin, irregular bowels, and quick hard pulse. At the orifice of the prepuce was a foul sloughy sore, completely surrounding it, discharging a glairy secretion.

Twelve leeches to be applied to the inflamed part, and afterwards Foment. Papav. c. Cataplasm.

℞. Liq. Antimon. Tartar. ʒjss.; Magnesiæ Sulph. ʒiij.; Liq. Ammoniæ Acet., Aquæ Menth. āā ℥iij. M. cap. coch. iij. mag. sextis horis.

Pil. Antim. Opiat. o. n. To keep his bed.

6th.—Slough beginning to extend, with increased pain of the sores; integuments thickened and hardened, with dark red inflammation. Tongue covered with thick white slimy fur; pulse 90—100, full and strong. Is very anxious.

7th.—Slough extending.

V.S. ad ℥xij.—Mist. Camph. ℥jss. c. Sp. Ætheris Sulph. ʒj. ter die. Pulv. Opii, gr. j. nocte maneque.—Catap. Theriac. Omitt. alia.

11th.—Pulse became much smaller and softer after the bleeding (on the 7th), and he expressed himself relieved: syncope was not induced. The sore remained stationary for two or three days; after which the slough again extended, and the pain increased.

Hirudines xij. pubis. Cap. Pulv. Opii, gr. j. ter quotidie.—Repet. alia.

From this time to the 16th, he rapidly improved. The slough separated, by which the glans was partially exposed. On the 14th he was found standing at the window, contrary to express order.

17th.—Considerable effusion into the integuments of the penis, with great induration, and dark red inflammation, made their appearance yesterday, attended with exquisite pain and extreme anxiety. The Acid. Nit. Fort. has been applied to-day. The pain produced by it was very severe, but lasted only twenty minutes; after which he became quite easy, and slept well for some hours.

18th.—Expresses much relief. The tumefaction has subsided, and the induration of the surrounding parts has abated. The subsidence of the swelling has exposed a foul sore at the frænum, situated on the glans; at which part only does he feel pain.

20th.—The slough has separated completely, leaving a healthy granulating surface. At the frænum, the sore is slowly spreading to the glans and integuments of the penis, being accompanied with a proportional increase of pain. His health is now much improved: tongue clean; bowels open; appetite good; sleep sound; pulse 70—80, moderately full and soft.

From this time the slough gradually and uninterruptedly spread to the surrounding parts, till, on the 25th, the whole surface of the original sore was contaminated; the tumefaction, induration, and pain, returning to the same extent as before.

26th.—The whole prepuce has sloughed off, and also a considerable portion of the glans, particularly about the frænum, where the urethra has been laid open about a third of an inch below its orifice; the urine passing in two streams,—one from this opening, the other from the orifice. Tongue covered with a thin white fur; slight thirst; bowels irregular; no appetite.

28th.—The whole glans, except a small portion at the upper part, in a sloughy state; as are the integuments of the penis for a considerable way down. Pain and anxiety excessive, so that he cannot remain quiet an instant.

Apply the Acid. Nit. Fort., and afterwards the soft Extr. Opii, reduced to the consistence of cream.

℞. Ammoniæ Subcarb., Moschi, āā gr. v.; Aquæ Menth. ℥ j.; Pulv. Opii, gr. j. M. sextis horis sumend.—Omit the former medicines.

The acid was applied in the evening, giving extreme pain, which abated in half an hour. He passed a good night, and remained quiet during the following day.

From this time, up to the 26th of October, little variation in the symptoms occurred, so that it is unnecessary to continue the daily reports. The slough, caused by the acid, separated in two or three days, but, as it fell off, the sloughing process again attacked the healthy surface left exposed, and continued its ravages upon the penis; its progress not being in the slightest degree impeded by the local applications or the medicines. The treatment pursued, as copied from the hospital-books, was as follows:

8th.—Apply the Unguent. Terebinth., instead of the Linim. Opii.

12th.—Omit the former medicines and applications, and substitute the following: ℞. Sulph. Quinæ, gr. iij.; Infusi Rosar. ℥ jss.; Tinct. Opii, m. x. sextis horis sumend.—Vini rubri, ℥ vj. quotidie.—Repet. Linim. Opii.—To be removed to a clean ward.

22d.—Omit the former medicines.—℞. Liq. Ammoniæ Acet. ʒ iij.; Misturæ Camph. ℥ j.; Spirit. Ætheris Nit. ʒ j.; Syrupi Papav. ʒ j. M. quartis horis sumend.—℞. Unguent. Cetac. ʒ vj.; Cerat. Plumbi, ʒ iij.; Pulv. Opii, ʒ j. fiat unguentum.—Ulceri applicand.

24th.—Omit the Syrupi Papav.—Cap. Ext. Conii, gr. iij. o. n. et m.—Foment. Papav. Tepid.—Cont. alia.

October 26th.—Sloughing continues to extend: it has now destroyed about two-thirds of the penis. Pain greater last night than it has been for some time past. Sleeps scarcely at all. Pulse 130, and weak; appetite bad; bowels open.

Sum. Pulv. Cinch. ʒj. secunda q. q. hora. Ext. Papav. gr. v. o. n.
Cont. Vini Rubri, ℥ vi. in die.
Argent. Nitr. ℈ii.; Aquæ Distill. ℥ j. fiat lotio ulceri applicanda. Over this let the saturnine and opium ointment, formerly ordered, be applied.
Omittantur alia.

31st.—Slough extending, the whole penis being now destroyed.

℞. Ext. Opii, ʒj.; Aquæ Bull. ℥ vj. fiat lotio tepid.—Cont. alia.

November 2d.—Pain of the sore unabated, which destroys his sleep. Some bleeding took place from the sore last night. Slough extending. Appetite continues good; bowels open; pulse 120.

Omitt. Cinchona.
℞. Hydr. c. Cretâ, gr. iv.; Pulv. Ipec. C. gr. iv. M. fiant pil. ij. sextis horis sumend.

5th.—Slough extending.
Omittantur medicamenta.
Extr. Sarsap. ʒ iij.; Decoct. Ejusd. ℔j. 3m. part. ter die.
Pulv. Ipec. C. gr. x. o. n.
Omitt. Unguent. Applic. Linim. Ærug.

9th.—Sloughing still extending.—Apply the nitric acid.

11th.—The application of the nitric acid gave him considerable relief, after the immediate pain resulting from it had subsided. The slough has separated; but the sloughing process has begun again at the edges of the sore, accompanied with a return of pain. Appetite bad.

℞. Ext. Cinchonæ, ℈ss.; P. Opii, gr. j. quartis horis sum.
Ext. Papav. gr. vi. o. n.—Omitt. alia.
Applic. Cetac. Simplex; and over this a poultice of bread and water.

14th.—Slough has extended to the whole front of the scrotum; but the upper part of the sore is quite clean, and covered with healthy granulations. There is no pain when dressed. Slept well the two last nights. Appetite improving; tongue moist and whitish.

November 19th.—Complains of pain in his back. Pulse 136, very weak; bowels confined. The sore is much deeper, and continues to extend: both testes are exposed, and their surface sloughy; the left one being completely denuded, except at the posterior part. The upper part of the sore healthy, and cicatrising. A slip of healthy granulations extends down between the testes.

Capiat Ol. Ricini, ʒ iv. statim.

21st.—Ulceration has again begun on the upper and left angle of the sore, where it was cicatrising. Since the testes have been exposed, he has complained much of the pain caused by their shaking, on the least motion of the body, and on this account expresses a great desire to have them removed.

30th.—The whole of both testes is denuded, except where their posterior part is in contact with the surface below, on which they rest.

Omitt. medicamenta.

℞. Ammoniæ Subcarb. gr. vij.; Infusi Cascaril. ℥ xj.; Tinct. Cinchonæ C. ʒ j.; Tinct. Hyoscyami, ʒ ss. M sextis horis sumend.

Cont. Ext. Papav.

December 3d.—Sore extending. Rest impaired; appetite bad; pulse 130, small and weak.

Omitt. Ext. Papav.
Cap. Pil. Sapon. c. Opio, gr. v. o. n. Vini Rubri, ℥ viij. in die.
Unguent. Hyd. ʒ j. brach. et cruribus infricetur mane et vespere.

7th.—Mercurial friction not begun till the 5th. No change of any kind, the mouth being not at all affected.

Infricetur Ung. Hydr. ʒ j. ter die.
Capiat Pil. Sapon. c. Opio, quarta quaque hora.
Let the Ung. Hydr. be applied to the sore.

8th.—Within ten minutes after the application of the ointment to the sore, he experienced great relief of the pain. He feels better to-day: his sleep last night was better than it has been for some time past; appetite is returning; mouth not at all affected.

10th.—Mouth not affected; dozes much; sore same as last reported; pulse 120, stronger; tongue clean.

Cerevis, ℔j. quotidie. Repet. medicamenta, &c.

14th.—Sore rather larger and deeper. Yesterday he was first observed to cough: he has no pain in the chest, and the expectoration is very slight. Appetite worse. Has little rest, and what he does obtain consists rather of dozing than proper sleep. Mouth unaffected.

Omitt. Frictio Mercurialis.—Repetantur alia.

17th.—Sore increases: the urethra and corpora cavernosa projecting above the level of the rest of the sore, and sloughy.

19th.—Unable to pass his urine. On a catheter being introduced a short distance into the urethra, and again withdrawn, he was enabled to empty the bladder. Pulse 130, excessively small and weak; tongue clean; sore extending; emaciation and anxiety extreme.

28th.—Gradually sunk, and died this morning, there having been no change in the symptoms since the last report. His cries, during the last five days, except when he was under the influence of opium (of which he took one grain every two hours), were so distressing as to disturb the whole ward.

*Examination.*—The sloughing surface covered the whole of the lower part of the abdomen, the upper edge of it extending across from the anterior superior spinous process of the ileum on the left side, to within one inch and a half of that on the right. There were also included within its limits the inner and fore part of each thigh, for about three inches downward. The surface presented an uniform level appearance, except where broken by the projection of the testes and corpora spongiosa and cavernosa of the penis. The testes were retained in their position solely by the spermatic cord; the remains of the penis projecting about two-thirds of an inch above the surrounding surface: both were sloughy, and, but for their prominence, could not have been distinguished from the rest of the sore.

The viscera of the abdomen, with the exception of the liver, which was a little larger than natural, were perfectly healthy. The glands within the abdomen and pelvis were not at all enlarged.—The head was not examined.

This case presents a melancholy instance of phagedenic ulceration, running on for many weeks, and resisting the various remedies, whether general or local, which were administered. It appeared from his own testimony, that this unfortunate lad had connexion once, and once only, with a female in Swan-alley. We understand that there are various houses of ill-fame in that vicinity, supplied with girls who are kidnapped about London-bridge, and other great avenues to the town, and who are afterwards liberally provided with spirits by their employers. These houses are frequented by foreign sailors; and it is probable that the very severe forms of sloughing ulcer, which have for some time attracted so much attention at St. Thomas's and other Hospitals in the City, are produced by the infection communicated by them to girls, most of whom are very young, and whose constitutions are impaired by mental depression and habitual intemperance.

